# Phase–Amplitude Coupling and Epileptogenic Zone Localization of Frontal Epilepsy Based on Intracranial EEG

**DOI:** 10.3389/fneur.2021.718683

**Published:** 2021-09-09

**Authors:** Huijuan Ma, Zeyu Wang, Chunsheng Li, Jia Chen, Yuping Wang

**Affiliations:** ^1^Department of Neurology, Xuanwu Hospital, Capital Medical University, Beijing, China; ^2^Department of Biomedical Engineering, Shenyan University of Technology, Shenyang, China; ^3^Beijing Key Laboratory of Neuromodulation, Beijing, China; ^4^Center of Epilepsy, Beijing Institute for Brain Disorders, Capital Medical University, Beijing, China

**Keywords:** electrocorticography, frontal lobe epilepsy, phase-amplitude coupling, modulation index, epileptogenic zone

## Abstract

**Objective:** This study aimed to explore the characteristics of phase-amplitude coupling in patients with frontal epilepsy based on their electrocorticography data, in order to identify the localization of epileptic regions and further guide clinical resection surgery.

**Methods:** We adopted the modulation index based on the Kullback-Leibler distance, phase-amplitude coupling co-modulogram, and time-varying phase-amplitude modulogram to explore the temporal-spatial patterns and characterization of PAC strength during the period from inter- seizure to post-seizure. Taking the resected area as the gold standard, the epileptogenic zone was located based on MI values of 7 different seizure periods, and the accuracy of localization was measured by the area under the receiver operating curve.

**Results:** (1) The PAC in the inter- and pre-seizure periods was weak and paroxysmal, but strong PAC channels were confined more to the seizure-onset zone and resection region. PAC during the seizure period was intense and persistent, but gradually deviated from the seizure-onset zone. (2) The characteristics of coupling strength of the inter- and pre-seizure EEG can be used to accurately locate the epileptogenic zone, which is better than that in periods after the beginning of a seizure. (3) In an epileptic seizure, the preferred phases of coupling were usually in the rising branches at the pre- and early-seizure stages, while those in the middle- and terminal-seizure were usually in the falling branch. We thus speculate that the coupling occurred in the rising branch can promote the recruitment of abnormal discharge, while the coupling occurred in the falling branch can inhibit the abnormal discharge.

**Conclusion:** The findings suggest that the phase-amplitude coupling during inter- and pre-seizure is a promising marker of epileptic focus location. The preferred phase of coupling changed regularly with the time of epileptic seizure, suggesting that the surge and suppression of abnormal discharges are related to different phases.

## Introduction

Frontal epilepsy accounts for about 20–30% of all kinds of partial epilepsy, and if long-term drug treatment is ineffective, we need to consider surgical treatment. However, the most important reason for the overall poor efficacy of frontal epileptic surgery is the difficulty in locating epileptic foci. Therefore, localization of epileptic foci is an important clinical problem. In recent years, phase–amplitude coupling (PAC) has been paid more attention to for localization of epileptogenic foci.

Brain rhythm variability has been detected across various temporal and spatial scales in both cortical and subcortical structures (1). This occurs not only in distinct brain states including wakefulness, drowsiness, and sleep (2), but also in neurological disorders (3) such as epileptic seizures (4). The brain rhythms of different frequency bands interact and regulate by themselves (5). The modulation of the amplitude of high-frequency oscillations (HFOs) by the phase of low-frequency oscillations (LFOs) is called PAC, which has been recommended as a potential biomarker for epileptic seizure and termination (6).

Until recently, there have been some exploratory researches in PAC of epilepsy. It was confirmed that the PAC during the inter-seizure period is helpful for localization of epileptogenic zone (7). Campora found that the epileptogenic regions had strong PAC before seizure, but decreased within the short period (8). Pasquetti discovered that the PAC in delta frequency between the neocortex and the CA1 region was enhanced during the inter-seizure period in rats with temporal lobe epilepsy (9). Gagliano studied dogs with bilateral intracranial electrodes implanted and found significant differences in the PAC between pre-seizure and inter-seizure. PAC in the epileptic foci was prominently high in the pre-seizure period, especially in the high-gamma band (10). A previous study of our group found that the “fall-max” pattern of PAC in the middle stage of epilepsy, which indicates that high-frequency amplitudes were the largest at the falling edges of LFOs, was a reliable biomarker for the location of temporal lobe epilepsy (11).

Although the relationship between PAC and epilepsy has become a research focus, there is still a lack of comparative studies on the characteristics of PAC in different stages of frontal epilepsy. To address these issues, our study collected the ECoG data from 12 patients with frontal lobe epilepsy. We calculated the MI based on the Kullback-Leibler distance (12), PAC co-modulogram, and time-varying phase-amplitude modulogram (13) to explore the temporal and spatial characterization of PAC strength and patterns during the period from inter- seizure to post-seizure.

## Methods

### Subjects

Nineteen seizures were obtained in the form of electrocorticography (ECoG) recordings from 12 patients with intractable frontal lobe epilepsy. The informed consent of each patient was obtained and approved by the ethics committee of Xuanwu Hospital. All patients underwent lesion resection at Xuanwu Hospital. Of the 12 patients, 10 has a good prognosis (Engel I) for more than 1 year, and two had a general prognosis (Engel III). The pathology of 9 cases revealed focal cortical dysplasia (FCD), 1 case was FCD with heterotopic gray matter, 1 case was cortical deformity and 1 case was tuberous sclerosis syndrome. The clinical characteristics of the patients are presented in Table 1.

**Table 1:** Sample characteristics.

**Subject**	**Sex**	**Age (years)**	**Epilepsy duration (years)**	**Seizure semiology**	**PET**	**MRI**	**MEG**	**lateralization**	**Resection location**	**Histopathology**	**Prognosis (engel grade)**
A_1,2,3_	M	7	4	GTC	Right OL and both TL	Right TL	Right FL and both PL	Right	Lateral frontal	FCD I	I
B	M	5	3	GTC	_	Right FL	Left FL	Right	Lateral and base of frontal	FCD II	I
C_1,2_	F	16	14	(1) SPS (2) GTC	(SPECT)Right FL,PL, TL, OL, Cerebel-lum	Right H	Mainly in right FL, some in left FL.	Right	Lateral frontal	FCD I	I
D_1,2,3_	M	12	6	(1)EMA (2) GTC	Right FL	The supratentorial ventricle was slightly dilated	_	Left	Lateral and base of frontal, cingulate	FCD I	I
E	F	9	3	(1) SPS (2) GTC	Normal	Normal	_	Right	Lateral frontal	FCD I	III
F_1,2_	M	28	17	(1) EMA (2) GTC	_	Both FL	Both TL	Left	Lateral and base of frontal, cingulate.	FCD I	I
G_1,2_	F	27	16	(1) SPS (2) GTC	Left TL	Normal	Both FL	Left	Lateral frontal	FCD I	I
H	Ftical	24	6	(1) SPS (2) GTC	-	Normal	Left PL	Left	Lateral frontal	FCD I	I
I	F	26	14	GTC	Left TL	Left FL	Right FL	Right	frontal	FCDII With heterotopic gray matter	I
J	M	28	20	GTC	_	Left FL	_	Left	frontal	cortical deformity	I
K	F	8	4	GTC	_	Normal	_	Left	frontal	FCDII	I
L	F	8	7	Myoclonus.	_	Both FL and PL, Right TL	_	Right	frontal	Tuberous sclerosis syndrome	III

*Twelve patients are marked by English letters. The Arabic numerals in the right of English letter indicate the number of seizure*.

*GTC, secondarily generalized tonic-clonic seizures; EMA, excessive movement automatism; SPS, simple partial seizures; FCD, focal cortical dysplasia; FL, frontal lobe; PL, parietal lobe; TL, temporal lobe; OL, occipital lobe; H, hippocampus*.

### ECoG Data Collection

The ECoG electrodes, made of 3.0 mm diameter platinum, were implanted on the cortex of each patient and were positioned 10.0 mm apart center-to-center according to clinical needs. The scalp Cz (the central of the head) was used as the reference electrode. Recordings were accompanied by video EEG monitoring (PN-NET, Yunshen Technology, Beijing, China). The sampling frequency of the ECoG data was 2048 Hz. The power-line interference was FIR notch-filtered at 50 Hz with all associated harmonics up to 450 Hz. The interfering channels were removed.

The seizure-onset zone (SOZ), Seizure onset (Tso) and seizure termination (T_ST_) were identified by epileptologists. The SOZ was defined by initial ECoG changes, which were characterized by sustained rhythmic discharges or repetitive spike-wave discharges that could not be explained by state changes and resulted in habitual seizure symptoms similar to those reported in previous studies. The resection area was determined based on clinical factors, including the irritative zone on ECoG, the extent of abnormality on MRI, and other non-invasive evaluation features.

We analyzed the signals from three seizure stages (8, 11), including the inter-seizure 300-s period (IS_300_: at least 2 h before seizure), 300 s pre-seizure (PS_300_: 300 s before the Tso), and the seizure period (from 60 s before the Tso to 60 s after the T_ST_). We, respectively, choose 10-s period with strong coupling from IS_300_ and PS_300_ and named it as IS and PS, and further identified five important periods during the seizure, including the pre-seizure (PS_10_: 10 s before the Tso of the seizure), the early-seizure (10 s after the Tso of the seizure), the mid-seizure (10 s in the middle of the seizure), the terminal-seizure (10 s before the T_ST_ of the seizure) and post-seizure (10 s after half a minute of the T_ST_). Finally, we obtained seven periods for each episode: IS, PS, PS_10_, early-seizure, mid-seizure, terminal-seizure and post-seizure.

The ECoG data were exported as European Data Format Plus (EDF+) files and imported into EEGLAB software to change the file format to Brainstorm. The raw ECoG signal was processed using the Brainstorm and MATLAB software (v7.10.0).

### Modulation Index

A modulation index (MI) was computed as a measure of cross-frequency coupling (12), which computed the amplitude by which a higher frequency is associated with the phase of a lower frequency. The phase frequencies (*f*_P_) were defined from 0.5 to 10 Hz in 0.1-Hz increments; the amplitude frequencies (*f*_A_) were defined from 13 to 200 Hz in 1-Hz increments.

The MI comodulogram was computed in 10-s windows that were shifted by 10 s. This allowed five cycles of the lowest rhythm (0.5 Hz) to be captured, while maintained a sufficient degree of continuity in the time domain.

### Statistical Analysis

The MI values of each electrode channel among different seizure periods were compared using the Kruskal–Wallis *H*-test and the multiple comparison test to identify significant differences. MI values between the electrode channels inside and outside the resection margin were compared using the Kruskal–Wallis *H*-test. The area under the curve (AUC) of receiver operating curve (ROC) for each period was used to measure the accuracy of localization of epileptogenic zone based on the MI values. The mean values of AUC for all episodes at 7 different periods were compared using the Kruskal–Wallis *H*-test and the multiple comparison test to identify significant differences.

## Results

### Temporal Specificity of Coupling

**Figure 1:**
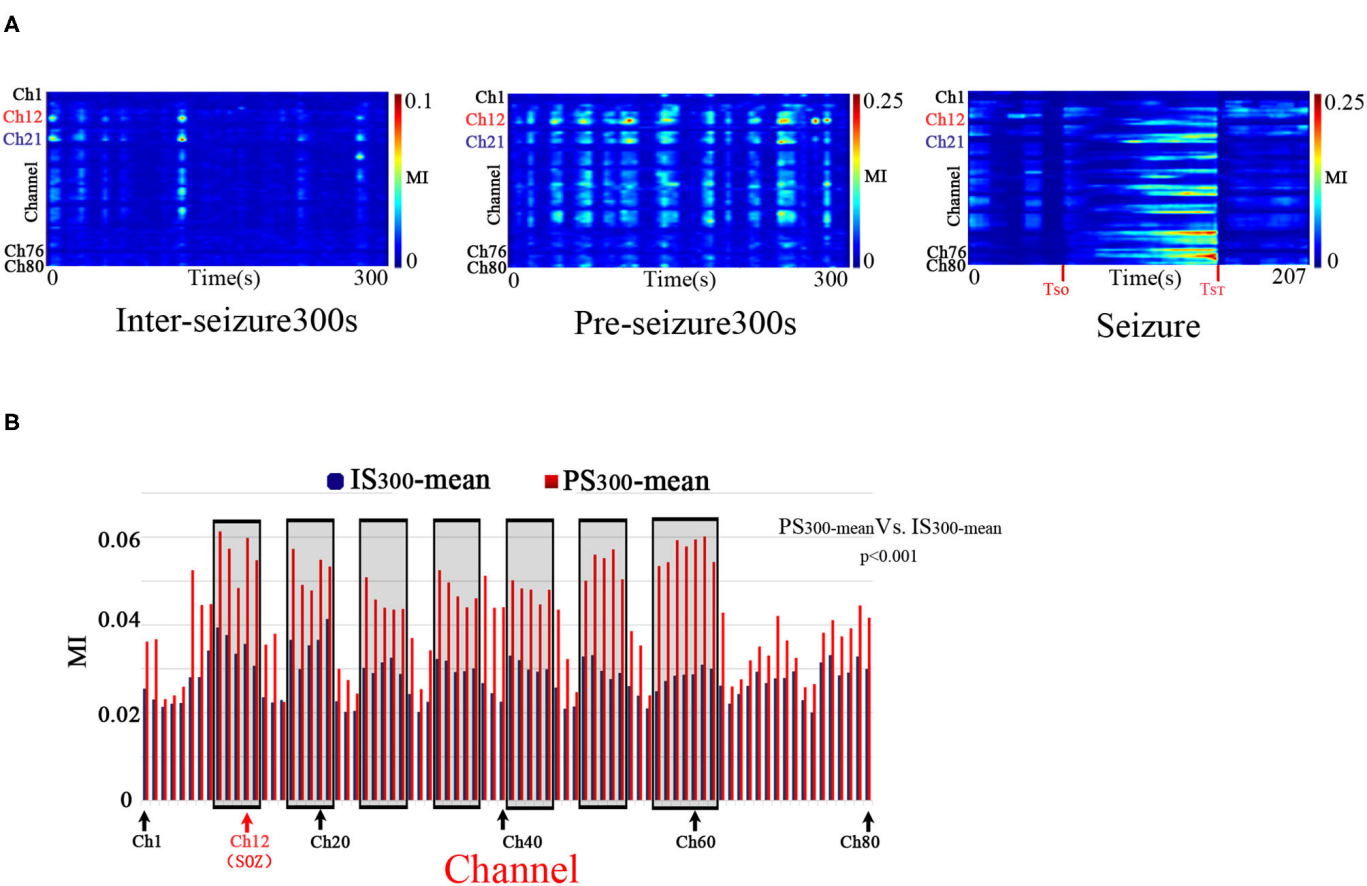
Temporalspecificity of PAC. **A:** The characteristics of the PAC at different stages. Modulation was computed in 10-s windows shifted by 10 s for all 80 electrode channels of Patient A across the IS_300_, PS_300_, and seizure recordings. The PAC rhythmically bursted in the IS_300_ and PS_300_ and continuously evolved during the seizure period. The PAC of IS_300_ was rare, weak, and brief, while the PAC of PS_300_ was rich, strong, and durable. The channel 12 (Ch12) was located in the SOZ (marked red); Ch21 was inside the resection margin but not in SOZ (marked blue); Ch1,76,80 was not in the resection margin and SOZ (marked black). **B:** Comparison of MI-mean values of each channel between the IS_300_ and PS_300_ period. The blue and red columns represent the MI mean-value of each channel during the IS_300_ and PS_300_ period, respectively. The black rectangles represent the resected area.

PAC was observed at the stages of IS_300_, PS_300_, and seizure in all patients. The PAC rhythmically burst in the inter- and pre-seizure periods, and continuously evolved during the seizure period. The inter-seizure PAC was rare, weak, and brief, while the pre-seizure PAC was rich, strong, and durable. Figure 1A shows the evolution of the PAC in different stages of Patient A.

The PAC strength in the resection margin of the PS_300_ was significantly higher than that of IS_300_, as measured by the MI-mean value of MI of each channel at 300 s between the IS_300_ and PS_300_ periods (*p* < 0.001, Kruskal–Wallis-test) (Figure 1B). The MI value was computed in 10-s windows that were shifted by 10 s, and the 30 MI values obtained were then averaged as MI-mean.

The strength, frequency and preferred phase of the PAC varied over time. The strongest PAC occurred in different channels at different times. And also, the MI values of each channel among the different seizure periods were significantly different (*p* < 0.001, Kruskal–Wallis-test and multiple comparison test). These results indicate a temporal specificity of PAC.

**Figure 2:**
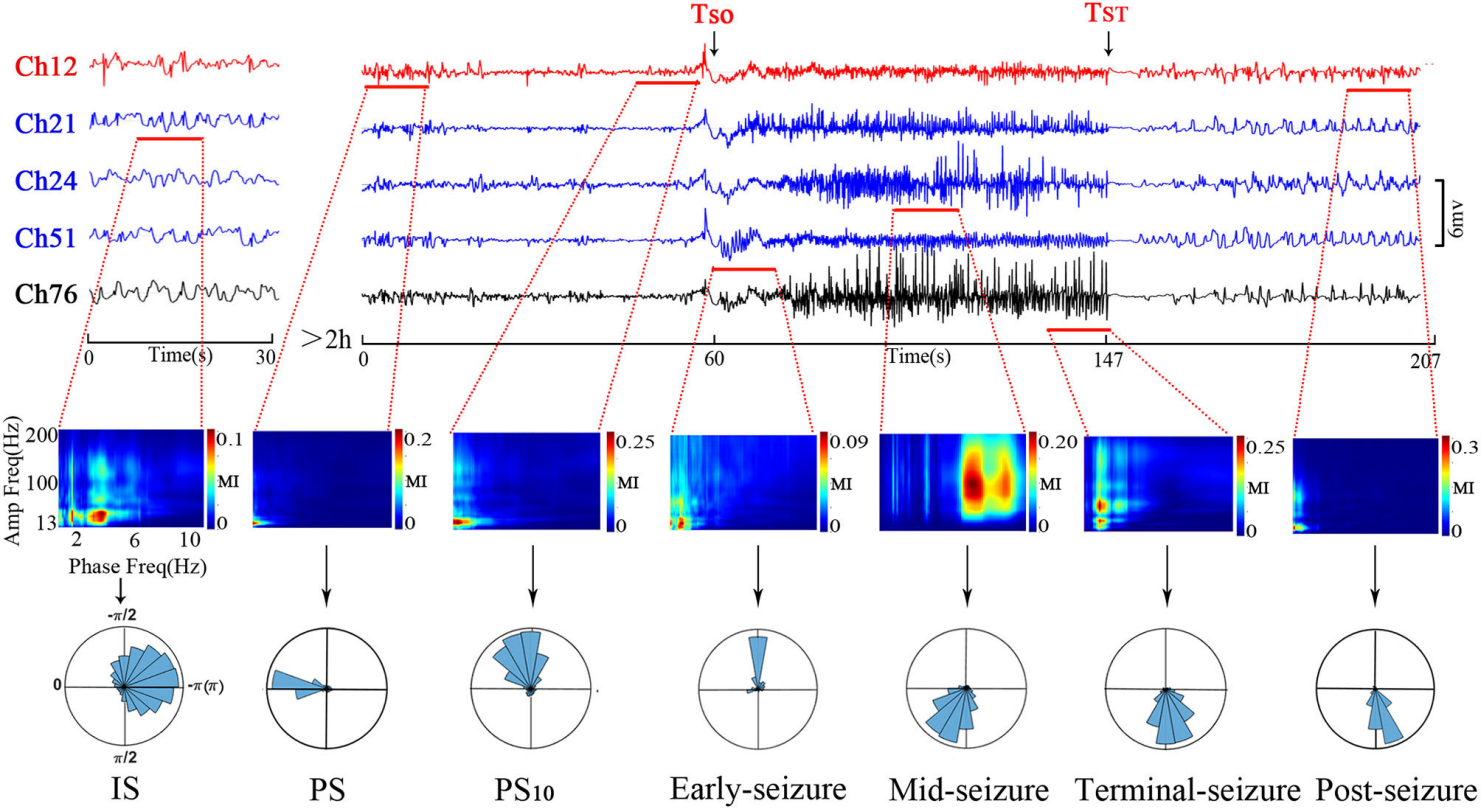
Temporal specificity of the PAC. The top panel shows channels that had the strongest PAC during the selected period. The channel 12 (Ch12) was located in the SOZ (marked red); Ch21, Ch24, and Ch51 were inside the resection margin but not in SOZ (marked blue); Ch76 was not in the resection margin and SOZ (marked black). The time intervals were shown below each trace. The ECoG segments with the most strongest PAC of the periods were marked by the red horizontal line. The most strongest PAC varied in different channels at the different period. The MI co-modulation of these ECoG segments are shown in the middle panel. In the middle panel, all axis labels are indicated on the leftmost sub-figure, and the scales are indicated by the color bar on the right side of each sub-figure. We found that the strength and frequency of PAC varies over time. The phase-amplitude polar histograms of these ECoG segments are shown in the below panel. We found that the preferred phases in the IS, PS, PS_10_, and early-seizure periods were in the rising phase (–π~0), while in the Mid-seizure, Terminal-seizure and Post-seizure periods, it was in the falling phase (0~π).

The PAC evolution of Patient A is shown in Figure 2. As shown in the top panel of Figure 2, the ECoG segments with the strongest PAC (marked by the red horizontal line) varied in different channels at the different period. As shown in middle panel of Figure 2, the low frequency of coupling varied predominently in the δ range, with the lowest frequency in the pre-seizure period, and increased to the θ-β range in the mid-seizure period. The high-frequency of coupling occurred in β-γ before the seizures onset and extended to the β-γ- ripple after seizure onset. As shown in below panel of Figure 2, the preferred phase of the LFOs in the IS, PS, PS10s and early seizure periods, at which the high-frequency amplitude maximum occurred, was generally located in the rising (–π~0) and peak phases, while in the Mid-seizure, Terminal-seizure and Post-seizure periods, it was located in the falling phase (0~π).

**Figure 3:**
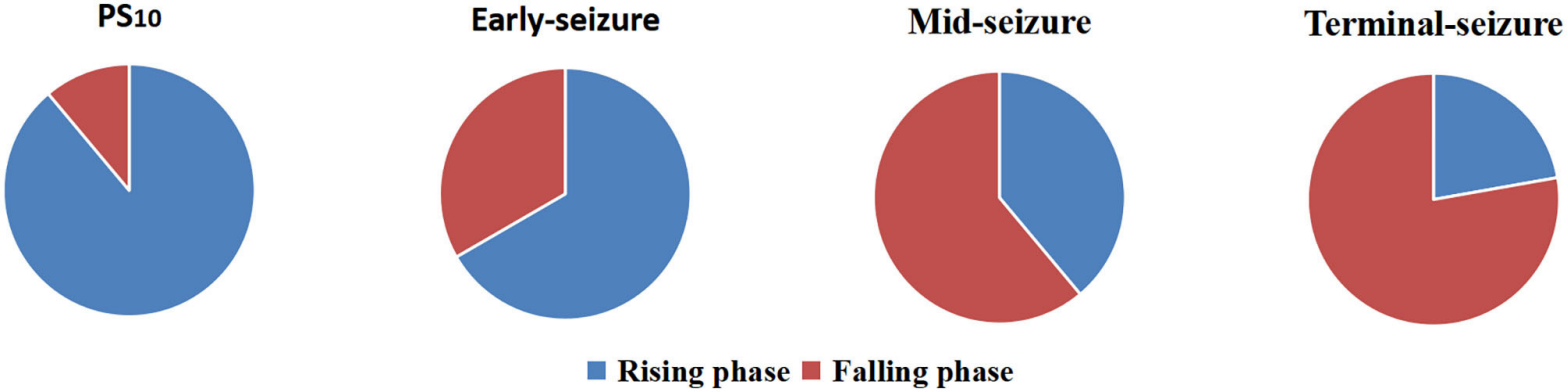
Statistics of preferred phases at four different periods of 18 episodes. The result showed that the preferred phases of coupling were usually in the rising branch at the PS_10_ and early-seizure, while that of the mid- and terminal-seizure were usually in the falling branch.

The phase-amplitude polar histograms of 18 seizures with duration longer than 20 s were calculated for 12 patients. As shown in Figure 3, the preferred phase of PS_10_ was mostly located in the rising branch (16/18), the preferred phase of early-seizure was mostly located in the rising branch and peak (12/18), the preferred phase of mid-seizure tended to locate in the falling branch (11/18), and the preferred phase of terminal-seizure was mostly in the falling branch (14/18).

### Spatial Specificity of EEG Dynamic Complexity

**Figure 4:**
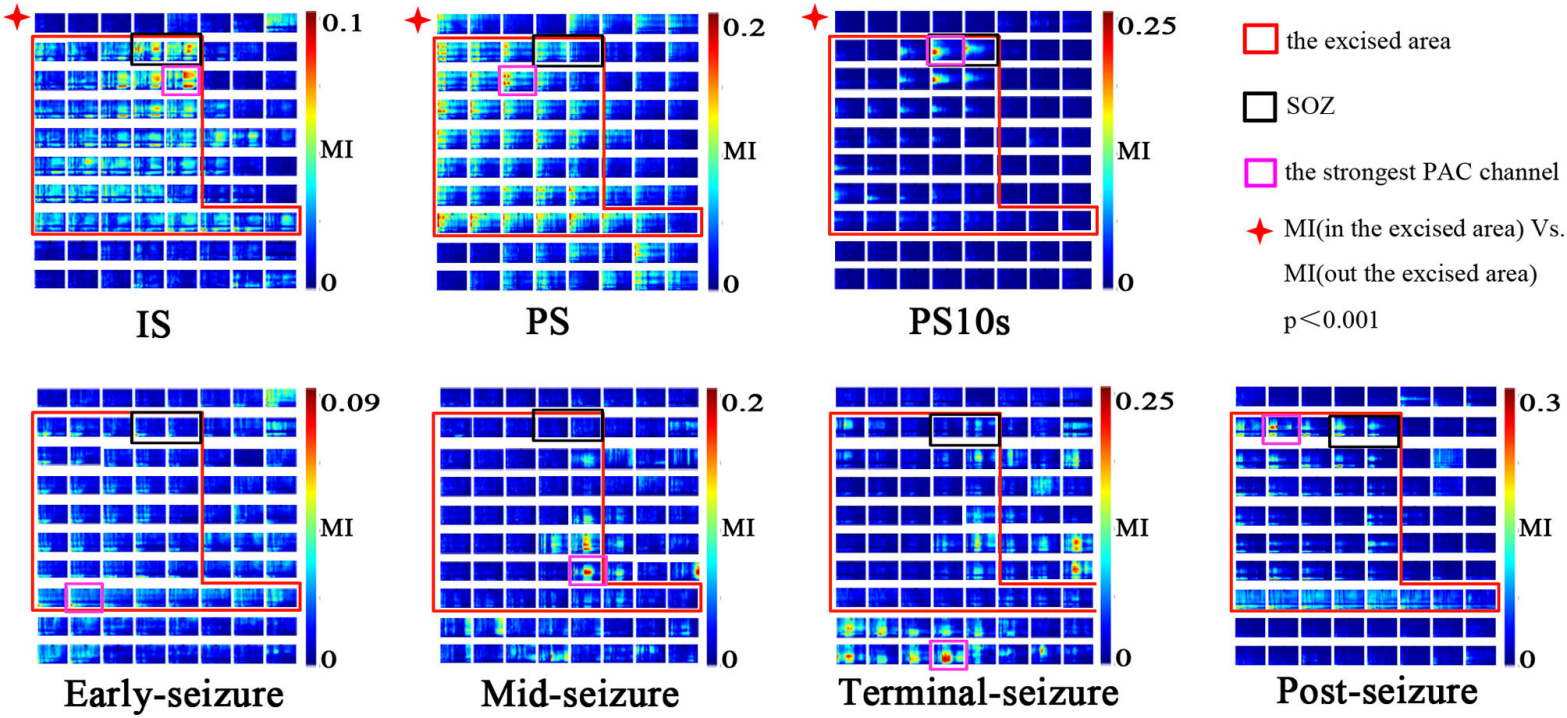
Spatial specificity of PAC. Modulation was computed in 10-s windows at different seizure periods (IS, PS, PS10, early-seizure, mid-seizure, terminal-seizure, post-seizure). The MI co-modulograms of all channels were arranged in turn in each sub-figure. The scales were indicated by the color bar on the right side of the sub-figure. The red rectangle is the excised area; The black rectangles is SOZ; The purple rectangles is the strongest PAC channel. This figure shows that the strong PAC in the IS, PS, PS10 periods was more concentrated on the resection margin. Once seizure begined, the strong PAC gradually subsided from SOZ to surrounding general resection area, and gradually translocated to the unresection area. In the post-seizure period, the strong PAC channels returned to the resected area. During the IS and PS period, the strongest PAC channel was usually located in resection margin very near but not SOZ; in the PS_10_, it was often located in the SOZ.

As shown in Figure 4, the channels in the resection area had strong PAC in the three periods of IS, PS and PS_10_, which were significantly higher than that in the unresection area (*p* < 0.001, Kruskal-Wallis test). After the onset of epileptic seizures, during the early-seizure, mid-seizure, and terminal-seizure periods, the channels with strong PAC gradually deviated from the SOZ, spread to the surrounding general resection area, and gradually translocated to the unresection area, and continued until the seizure termination. After a seizure, in the post-seizure period, the strong PAC return to the resected area.

During the IS and PS period, the strongest PAC channel was usually located in the resection margin near SOZ, while it was often located in the SOZ during the PS_10_ period. As shown in Figure 4, the purple rectangles is the strongest channel while the black rectangles is the SOZ. The two site were similar but not consistent at the IS and PS period, while consistent at the PS_10_ period.

The 7 subgraphs in Figure 4 are the MI comodulograms of all channels at 7 different periods arranged in turn of patient A. The coupling frequencies of Is, PS, PS_10S_, early-seizure, post-seizure were mainly distributed over 13–60/0.5–4 Hz. The MI comodulograms of this frequency range were thus shown for the five periods. The coupling frequencies of Mid-seizure and Terminal-seizure were mainly distributed over 13–160/0.5–4 Hz, the MI comodulograms of this frequency range were thus shown in both the periods.

**Figure 5:**
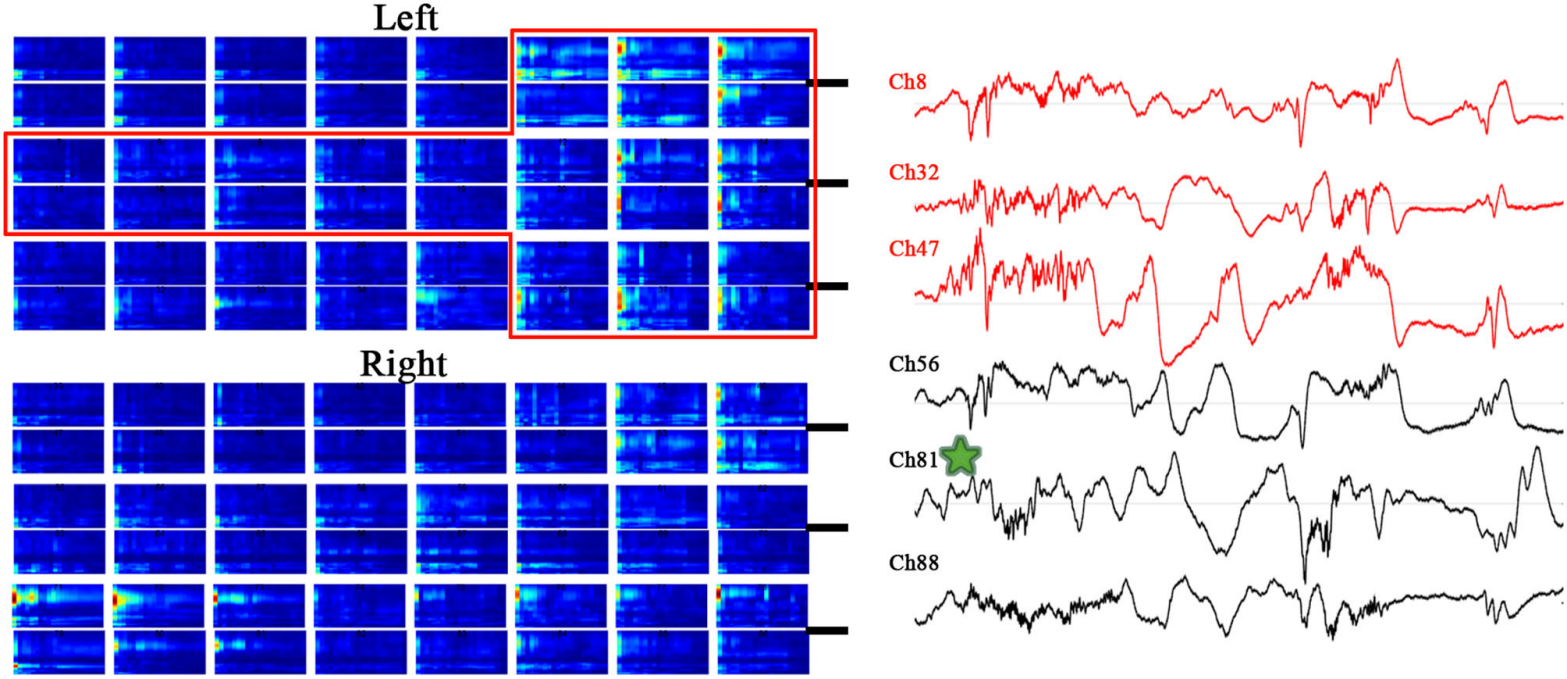
MI co-modulogram and original EEG during PS_10_ of special case F. The red rectangle is the excised area; the strongest PAC channel is mark by green star. Although some strong PAC channels on the contralateral cortex of the resection area, the strong PAC channels in the contralateral cortex were not centralized than that in the resection area, and the slow wave of raw ECoG on the contralateral cortex was more significant than that on the resection side.

Although some cases had strong PAC channels in the contralateral cortex of the resection area, combined with the concentration of the strong PAC channels, we could still accurately locate SOZ. Such as the case F, the SOZ of case F is located near the midline of the brain in the basilar and medial frontal lobes. During the IS and PS, the mirror position of SOZ on the opposite side also had obvious PAC, and even the strongest coupling channels was in the contralateral CH81. But the strong PAC channels in the contralateral cortex were not centralized than that in the resection area, and the slow wave of raw ECoG was more significant on the contralateral cortex than that on the SOZ side (Figure 5). According to these detailed features, the epileptic area can still be accurately located.

### Epileptogenic Zone Localization Based on MI Values

**Figure 6:**
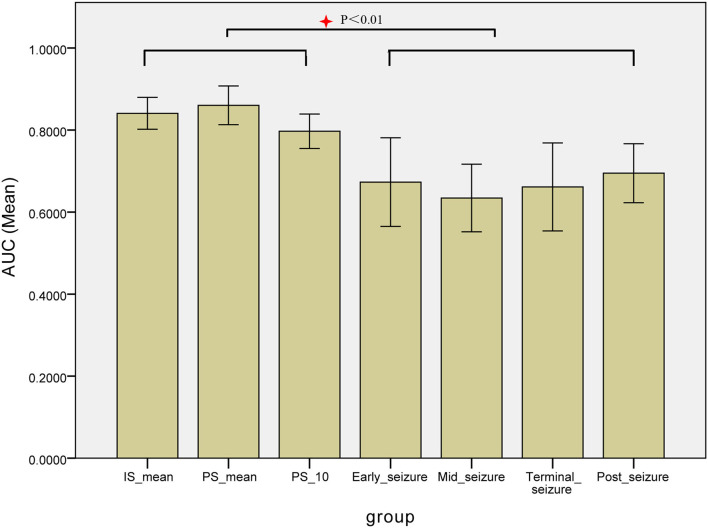
Comparison the AUC of the ROC for each period of the localization based on the MI values. The AUC of the ROC for each period was used to measure the accuracy of the localization based on the MI values of each patient at 7 periods from IS_300_ to post-seizure, respectively, The localization based on the MI-mean value of PS_300_ was the most accurate, followed by MI-mean value of IS_300_ and PS_10_. The three MI values were better than other periods, and there was no statistical difference among IS_300_, PS_300_, and IS_10_.

The epileptogenic zone localization based on the MI values of each patient at 7 periods from IS_300_ to post-seizure, respectively, and the MI values of IS_300_ and PS_300_ were, respectively, used the MI-mean value of the entire 300-s ECoG. The AUC of the ROC for each period was used to measure the accuracy of the localization. The results showed that the localization based on the MI-mean value of PS_300_ was the most accurate, followed by the MI-mean value of IS_300_ and PS_10_. The three MI values were better than other periods from early-seizure to post-seizure (*P* < 0.01, Kruskal-Wallis test), and there was no statistical difference among IS_300_, PS_300_, and PS_10_ (*P* > 0.3, Kruskal-Wallis-test). The results are shown in Figure 6 and Table 2 (The form of Seizure L was myoclonus, the duration was very short, and there was no evolution of Seizure period, so the AUC from PS10 to terminal-seizure could not be calculated).

**Table 2:** Comparison the AUC of the ROC for each period of the localization based on the MI values.

	**IS_300_-mean**	**PS_300_-mean**	**PS_**10**_**	**Early-seizure**	**Mid-seizure**	**Terminal-seizure**	**Post-seizure**
Seizure A1	0.997	0.956	0.813	0.715	0.305	0.267	0.915
Seizure A2	0.989	0.983	0.902	0.835	0.888	0.735	0.797
Seizure A3	0.984	0.971	0.855	0.790	0.831	0.737	0.844
Seizure B	0.892	0.894	0.682	0.344	0.901	0.746	0.794
Seizure C1	0.783	0.839	0.858	0.587	0.599	0.725	0.848
Seizure C2	0.757	0.858	0.678	0.714	0.681	0.660	0.837
Seizure D1	0.850	0.925	0.785	0.883	0.746	0.804	0.729
Seizure D2	0.781	0.785	0.803	0.943	0.691	0.886	0.699
Seizure D3	0.770	0.784	0.882	0.913	0.445	0.480	0.574
Seizure E	0.767	0.805	0.768	0.388	0.668	0.569	0.528
Seizure F1	0.860	0.920	0.868	0.597	0.488	0.740	0.570
Seizure F2	0.893	0.868	0.590	0.313	0.470	0.113	0.501
Seizure G1	0.768	0.855	0.831	0.866	0.627	0.730	0.425
Seizure G2	0.777	0.831	0.811	0.715	0.690	0.878	0.441
Seizure H	0.789	0.543	0.803	0.239	0.605	0.956	0.674
Seizure I	0.856	0.843	0.749	0.788	0.712	0.807	0.743
Seizure J	0.863	0.844	0.914	0.710	0.693	0.577	0.685
Seizure K	0.755	0.959	0.755	0.775	0.377	0.495	0.863
Seizure L	0.842	0.882	–	–	–	–	0.734
Mean ± std.	0.841 ± 0.081	0.860 ± 0.100	0.797 ± 0.084	0.673 ± 0.217	0.634 ± 0.166	0.661 ± 0.216	0.695 ± 0.149

## Discussion

In this study, we examined the spatiotemporal characteristics of the PAC based on ECoG signals collected from 19 seizures of 12 patients with frontal lobe epilepsy. The PAC was measured by MI comodulogram during different periods of seizures. The results suggest that the PAC during inter- and pre-seizure is a promising marker for epileptic focus location. The preferred phase of coupling changed regularly with the epileptic seizure.

The seizure period has attracted much attention because of its feature of significant and persistent PAC, while the inter- and pre-seizure PAC have been ignored because of their weak and brief nature. However, the strong PAC channels in the inter- and pre-seizure periods were more confined or close to the SOZ, and it can assist the localization of the epileptogenic zone. In the seizure, the strong PAC channels gradually deviated from the SOZ, and its usefulness for positioning weakened. The PAC strength of the resected area at PS_300_ was significantly increased, especially the channels in and around the SOZ, which provides strong support for using PAC for SOZ localization and epileptic seizure prediction.

It is particularly intriguing that the localization based on MI strength of inter-seizure can also achieve high accuracy, which is not statistically different from the pre-seizure localization. Since the ECoG of inter-seizure is easier to obtain, the long period of extraoperative ECoG recording needed to capture habitual seizures can be avoided.

We found that using the MI mean value of IS_300_ and PS_300_ to locate epileptic areas is more accurate than that of the MI of PS_10_, suggesting that long-range electroencephalogram could collect more coupling information and highlight the frequently-occurring strong coupling channels, which was better than the short-range electroencephalogram in epileptogenic zone localization.

The current view is that PAC facilitates effective interactions between neurons with similar phase preferences. The specific phase of LFOs modulates and promotes the amplitude of HFOs in tune surge (1), and strengthen the synchronization of HFOs (14). The LFO_S_ in epileptic seizures modulates and promotes abnormal discharge recruitment and propagation by the role of PAC. PS_10_ is the period of initial abnormal discharge recruitment, the PAC in SOZ is thus more stronger than the other channels. However, in IS and PS, abnormal discharge spreads in a small range near SOZ, so strong PAC is not only in the SOZ but also in the channels around SOZ, and the strongest PAC channel is often not completely consistent with the SOZ. Even in some special cases whose SOZ is near the mid-line of the brain and corpus callosum, where the conduction fibers are well-developed. The strongest PAC channel may appear on the opposite side of the SOZ during the periods of IS and PS. These findings reflect that PAC plays an important role in the propagation of epileptic discharge. However, because the abnormal discharge during IS and PS did not propagate widely from SOZ, but were confined to and around SOZ, the PAC in these two periods was more accurate to locate SOZ.

Previous studies have reported that pulse stimulation of the SOZ or cortico-thalamic tract can induce spike-and-slow-wave discharges in the surrounding regions (15, 16). This process is similar to the PAC around the SOZ during the inter- and pre-seizure periods. This supports our hypothesis that HFO_S_ occurring in SOZ is the initiator of PAC and causes the surege of HFO_S_ in the surrounding nerve cells through the macroscale neural ensembles of the brain, leading to the transmission of HFO_S_.

Many investigators have reported that the electrode sites that frequently generated HFOs often turn out to be a part of the SOZ (17–19). That is, the channels where the PAC frequently occurred are prone to trigger HFOs and are potential biomarkers for epilepsy. Our research also identified that most of strong PAC channels of the inter- and pre-seizure periods were located inside the resection margin and were important for a seizure-free prognosis after surgery, while part of the strong PAC channels in case E and L was unresected, and their prognosis was only Engel III, which further confirmed that PAC is a potential biomarker for epileptic seizures.

During the period from PS_10_ to early-seizure, HFOs preferentially took place at rising phase of LFO_S_. During the mid-seizure and terminal periods, HFOs preferentially occurred during the falling phase of LFO_S_. The finding suggest that the surge and suppression of abnormal discharges are related to different coupling phases.

In this study, we observed the frequency, phase and spatial characteristics of PAC in different seizure periods through intracranial EEG of frontal lobe epilepsy, but these phenomena need to be thoroughly studied in neurodynamics before they can be well-applied in clinical practice. In the future, more cases and technical methods are needed to further explore the underlying neurodynamics of these phenomena.

## Data Availability Statement

The original contributions presented in the study are included in the article/supplementary material, further inquiries can be directed to the corresponding author.

## Ethics Statement

The studies involving human participants were reviewed and approved by the Ethics Committee of Xuanwu Hospital, Xuanwu Hospital. Written informed consent to participate in this study was provided by the participants' legal guardian/next of kin.

## Author Contributions

YW participated in the design or conceptualization of study. HM in interpretation of data or drafting manuscript. ZW and CL in data analysis. JC in manuscript revision. All authors contributed to the article and approved the submitted version.

## Funding

This work was supported by National Natural Science Foundation of China (Grant No. 81771398).

## Conflict of Interest

The authors declare that the research was conducted in the absence of any commercial or financial relationships that could be construed as a potential conflict of interest.

## Publisher's Note

All claims expressed in this article are solely those of the authors and do not necessarily represent those of their affiliated organizations, or those of the publisher, the editors and the reviewers. Any product that may be evaluated in this article, or claim that may be made by its manufacturer, is not guaranteed or endorsed by the publisher.
